# Health Implications of Shift Work in Airline Pilots and Cabin Crew: A Narrative Review and Pilot Study Findings

**DOI:** 10.3390/nu17243906

**Published:** 2025-12-13

**Authors:** Oliwia Stefańska, Olga Barbarska, Anna Minkiewicz-Zochniak

**Affiliations:** 1 Student Scientific Club Agar, Department of Medical Biology, Medical University of Warsaw, Litewska 14/16, 00-575 Warsaw, Poland; oliwka.stefanska@wp.pl; 2 School of Medical & Health Sciences, VIZJA University, 59 Okopowa St., 01-043 Warsaw, Poland; o.barbarska@vizja.pl; 3 Department of Medical Biology, Medical University of Warsaw, Litewska 14/16, 00-575 Warsaw, Poland

**Keywords:** circadian misalignment, shift work, aviation medicine, airline pilots, cabin crew, nutritional behavior, metabolic health, occupational fatigue

## Abstract

**Background:** Airline pilots and cabin crew are exposed to multiple occupational stressors, including circadian disruption, irregular meal timing, cabin environment and radiation, which collectively affect sleep, metabolism and overall health. This study aimed to evaluate the health implications of shift work in aviation by combining self-reported experiences with existing scientific evidence. **Methods:** A cross-sectional survey of 101 airline personnel was conducted to assess sleep patterns, fatigue, nutrition-related challenges and health symptoms. The survey findings were integrated with a literature review to contextualize observed health outcomes within known effects of circadian disruption and aviation-related stressors. **Results:** Sleep disturbances (71%) and fatigue (89%) were the most prevalent symptoms, while 60% of respondents reported weight fluctuations and 50% limited access to nutritious food during duty. Appetite alterations, reduced taste perception and frequent melatonin use indicated behavioral adaptation to circadian misalignment. Among female aircrew (63%), thyroid and reproductive concerns were reported, aligning with documented impacts of radiation exposure and endocrine disruption. The findings correspond with existing evidence linking aviation-related circadian stress to cardiometabolic, endocrine and gastrointestinal imbalance. **Conclusions:** Shift work and occupational exposures in aviation contribute to significant disturbances in sleep, metabolism and overall health among aircrew. Preventive strategies should integrate fatigue risk management, circadian-aligned scheduling, improved in-flight nutrition and comprehensive occupational health surveillance to safeguard crew well-being and operational safety.

## 1. Introduction

Recent decades have witnessed a marked transformation in employment patterns, with a significant decline in standard eight-hour work schedules coinciding with a substantial increase in shift work prevalence. Shift work is widely recognized as a major disruptor of circadian homeostasis, defined as permanent or periodic professional activity outside the typical working hours (7:00–18:00), and is consistently perceived as atypical and burdensome [1,2]. Throughout Europe, shift workers currently represent approximately 21% of the labor force [3]. Among shift workers, pilots and cabin crew constitute a distinctive occupational group, numbering roughly half a million pilots and over one million cabin crew worldwide. Industry indicates the need to employ nearly one million additional aircrew members by 2034 due to fleet expansion and workforce replacement [4,5,6].

Shift work systems operate through either permanent schedule with fixed timing or rotating patterns that may progress clockwise, counterclockwise, or irregularly. Among these arrangements, rotating systems prove particularly burdensome, as they prevent the establishment of stable habits and impede bodily adaptation. Regardless of the model adopted, shift work leads to circadian rhythm disturbances, reduced sleep quality, and lifestyle modifications, including dietary habits [7]. Extensive research has demonstrated that pilots and cabin crew are particularly susceptible to sleep disorders, fatigue, daytime sleepiness, reduced sleep quality, and circadian rhythm disruptions, especially when working night shifts or on intercontinental routes [8,9]. Moreover, misalignment between internal circadian rhythms and external factors such as irregular meal timing or light exposure may compromise metabolic regulation. This phenomenon is central to the emerging field of chrononutrition, which examines how the timing of food intake interacts with circadian biology to influence health outcomes.

Among aircrew members, circadian rhythm disruptions trigger desynchronization of physiological processes while simultaneously increasing the prevalence of sleep disorders and metabolic consequences [10]. These disruptions have also been linked to adverse metabolic changes, including elevated body mass index [10,11]. Additionally, chronic fatigue and restricted exposure to daylight contribute to mood deterioration and heightened depressive symptoms [12,13], while reduced sleep quality and irregular working hours result in diminished alertness, concentration, and overall work capacity [14,15].

The unique occupational demands facing pilots and cabin crew encompass frequent time-zone transitions, extended periods in confined aircraft environments, and restricted access to nutritionally adequate food options. These challenging conditions result in irregular eating patterns, frequent snacking behaviors, excessive caffeine consumption, and compromised overall diet quality-specifically characterized by insufficient fruit and vegetable intake alongside elevated saturated fat consumption [16,17]. Meals are typically consumed in accordance with work breaks rather than biological rhythms, which contributes to nutritional imbalance and poor adherence to dietary recommendations, especially among women [16,17]. Preliminary research has also indicated a high prevalence of being overweight and limited compliance with dietary guidelines [18]. Occupational factors, such as long flight times and inflight catering limitations, further reinforce unfavorable patterns [19].

The combination of irregular dietary patterns and restricted access to healthy food options represents a significant risk factor for metabolic disorders, obesity, cardiometabolic complications, and diminished psychological well-being [8,16,19,20]. Furthermore, misalignment of meal timing with biological rhythms may intensify jet lag symptoms, heighten fatigue, and compromise sleep quality [7,21].

Given these multifaceted challenges, investigating dietary habits and their health implications among aircrew becomes essential not only for individual well-being but equally for occupational safety and public health considerations. The implementation of circadian-aligned dietary and organizational interventions could effectively mitigate the adverse effects of shift work and frequent time-zone travel [2,16,22,23].

However, it is important to recognize that nutrition constitutes merely one component within the complex array of factors determining pilots’ and cabin crew’s overall health. Shift work and aviation-specific environmental factors also contribute to numerous somatic and psychological risks described in the literature as serious hazards for this occupational group [8,9,24].

Considering the expanding demand for aviation personnel coupled with heightened awareness of shift work-related health challenges, developing a comprehensive understanding of occupational risks within this population becomes paramount. This paper integrates findings from a pilot survey among airline personnel with a narrative review of the literature to provide a broad perspective on the health implications of aviation work. By combining empirical data with existing evidence, we aim to identify key areas of concern and highlight strategies that may help mitigate the adverse consequences of shift work and aviation-specific exposures.

## 2. Materials and Methods

### 2.1. Study Design

This study employed hybrid research design, combining a cross-sectional survey with a narrative literature review. The purpose of this mixed approach was to integrate empirical data with existing evidence to provide a comprehensive overview of the health implications of shift work among airline personnel.

This pilot study was designed as an exploratory, non-hypothesis-driven investigation aimed at descriptively characterizing health- and nutrition-related patterns among aircrew. No a priori hypotheses were formulated, consistent with the observational and descriptive nature of the study.

### 2.2. Survey Component

The cross-sectional survey was conducted among airline personnel engaged in shift work, including both flight attendants and pilots. The study population consisted of 101 participants who met the inclusion criteria: age ≥ 18 years and current employment as either a flight attendant or a pilot. Recruitment was carried out using a purposive non-random sampling strategy. Participation was entirely voluntary and anonymous.

Data collection was based on the diagnostic survey method. A structured, self-administered questionnaire specifically developed for this study was used. The instrument was available in two language versions (Polish and English) and distributed electronically via a Microsoft Forms link. Respondents completed the survey independently.

The questionnaire consisted of 31 items, including single-choice, multiple-choice, Likert-scale and open-ended questions, covering the following thematic domains: (1) sociodemographic characteristics, (2) access to food and dietary behavior during flights, (3) appetite and taste perception changes, (4) eating patterns on duty vs. at home, (5) physical activity habits, (6) sleep quality and fatigue, (7) psychological well-being and stress, (8) chronic diseases and other health changes, (9) use of melatonin and dietary supplements, (10) awareness of occupational risks including cosmic radiation.

The questionnaire underwent expert content validation by two specialists in aviation medicine and two specialists in nutrition/public health, who assessed clarity, relevance, and coverage of pertinent health domains. Prior to data collection, the survey was pilot-tested on a group of 10 airline employees to ensure comprehensibility and accurate interpretation of items. Minor linguistic and structural adjustments were made accordingly. Due to the multidimensional character of the instrument, Cronbach’s alpha was not applicable; however, internal logical consistency between related items was confirmed during pilot testing.

To capture perceived health changes over the course of an aviation career, the questionnaire included items assessing whether participants noticed the onset of new conditions since entering the profession.

Items referring to chronic medical conditions (e.g., thyroid dysfunction, hypertension, chronic fatigue) were designed to capture only previously diagnosed conditions. Participants were instructed to report exclusively medically established diagnoses.

This study utilized descriptive statistical methods, consistent with its exploratory design and primary objective of characterizing health- and nutrition-related patterns within the study population. The analytical framework prioritized comprehensive description over hypothesis testing, reflecting the study’s foundational role in establishing a baseline understanding of population characteristics. Continuous variables are presented as means ± standard deviations, while categorical variables are reported as frequencies and percentages. All statistical analyses and data visualizations were performed using Microsoft Excel (Microsoft Corporation, Redmond, WA, USA).

This descriptive approach was deliberately selected to identify and characterize health and nutrition trends rather than to test predetermined hypotheses, thereby providing essential groundwork for future hypothesis-driven research.

### 2.3. Narrative Review Component

The narrative review was designed to provide a comprehensive synthesis of multidisciplinary evidence on the health consequences of shift work and aviation-specific environmental exposures among flight crew. The review integrates findings related to circadian disruption, metabolic health, cardiovascular risk, nutritional challenges, sensory alterations, respiratory and endocrine function, gastrointestinal and microbiome balance, and evidence-based preventive strategies. Consistent with narrative review methodology, the aim was to summarize representative and conceptually relevant evidence across key thematic domains rather than to exhaustively identify all studies containing individual keywords.

A structured non-systematic literature search was performed in the PubMed, Scopus, and Google Scholar databases using combinations of keywords and Medical Subject Headings (MeSH): shift work, circadian rhythm, pilots, flight attendants, aviation, nutrition, sleep, melatonin, cardiovascular disease, cancer, hormones, radiation, microbiome, and occupational disorders. The search strategy was designed to ensure adequate coverage of major concepts and multidisciplinary evidence, not full retrieval and screening of all records returned by each keyword. Only peer-reviewed articles in English published between 2003 and 2025 were included.

### 2.4. Ethics and Data Protection

This survey was anonymous and minimal risk, involving no intervention or collection of sensitive personal data. Participation was voluntary, and no identifying information (names, contact details, IP addresses) was collected. The study was conducted in accordance with the principles of the Declaration of Helsinki and complied with the general data protection regulations (GDPR). Data were securely stored in encrypted form and analyzed in aggregate to ensure full anonymity. Formal ethical approval was not required for this type of anonymous, non-interventional research, in accordance with institutional and national regulations.

## 3. Results

### 3.1. Study Population

A total of 101 aviation personnel participated in the study, comprising 84% flight attendants and 16% pilots, with 63% of participants being women. The majority were aged 18–35 (82%) and had a university degree (70%). A breakdown of work experience revealed 47% with 1–5 years and 34% with >5 years of aviation experience.

### 3.2. Occupational Health Outcomes

**Figure 1:**
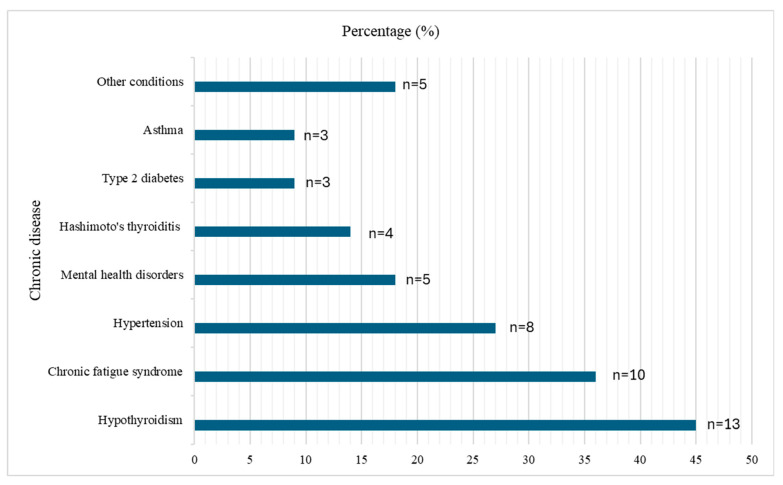
Prevalence of chronic diseases developing after starting work in aviation (*n* = 29).

Analysis of occupational health outcomes revealed pronounced sleep and circadian rhythm disturbances, with 71% of participants reporting sleep problems and 89% experiencing frequent work-related fatigue (including 62% who reported fatigue during work and 62% after completing their duties). Nearly one-third of respondents (29%) reported developing chronic diseases after starting their aviation career, with the most common conditions being hypothyroidism (45% of those affected), chronic fatigue syndrome (36%), hypertension (27%), and mental health disorders (18%). Weight changes were also common, affecting 60% of participants, with 38% reporting weight gain and 22% reporting weight loss after starting their aviation career (Figure 1). These changes were frequently accompanied by self-reported appetite alterations and irregular meal timing.

### 3.3. Dietary Behavior and Circadian Misalignment

Irregular working hours and circadian rhythm disturbances were frequently associated with changes in dietary behavior. Half of the respondents (50%) reported limited access to nutritious food during work shifts. Appetite changes were common, with 47% of participants reporting an increase in appetite and 21% reporting a decrease in appetite since beginning their employment in aviation.

**Figure 2:**
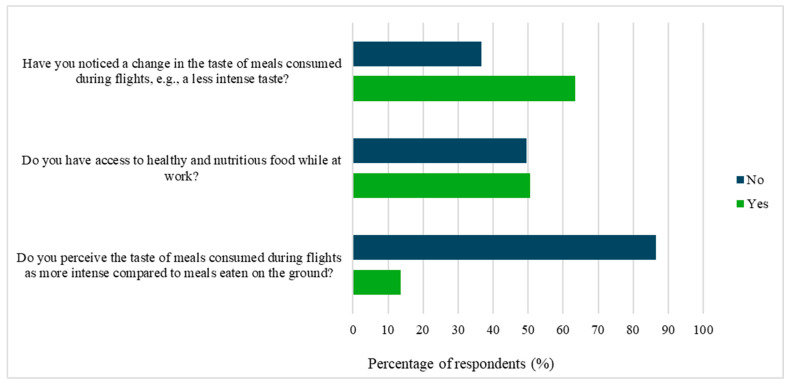
Perception of appetite and taste changes among flight and cockpit crew (*n* = 101). The figure presents responses regarding changes in appetite, access to healthy food, and perception of meal taste intensity during flights.

Altered taste perception was reported by 63% of participants, most often describing meals consumed during flights as having a less intense taste compared to those eaten on the ground (Figure 2). Only 13% of respondents perceived the taste as more intense. Meal structure also differed substantially between workdays and days off. During flights, the majority (57%) consumed airline catering meals, 29% brought homemade food, and smaller groups relied on ready-made or snack-type options. Over 80% of participants reported differences in meal timing or composition between workdays and days off.

### 3.4. Supplement Use and Self-Management

Melatonin supplementation was reported by 41% of participants, with 76% of them noting improved sleep quality and reduced difficulty falling asleep. The use of other dietary supplements was declared by 56% of participants. The most common were vitamin D (50% of supplement users), multivitamin complexes (43%), omega-3 fatty acids (43%), and magnesium (43%). Fatigue reduction and mood improvement were the primary motivations for supplement use.

Health literacy was high: 85% of participants were knowledgeable about the risks associated with exposure to ionizing radiation, and 92% were aware of the general health consequences of working in aviation. Despite this, only 50% reported adequate access to nutritious food during their workday. Interest in occupational health consultation services was significant: 55% expressed willingness to participate in preventive medical consultations, 23% were not interested, and 22% expressed uncertainty.

## 4. Occupational Risks and Health Consequences in Aircrew: A Narrative Review

Aviation professionals encounter a unique constellation of occupational factors that synergistically interact to influence multiple physiological systems. These factors include environmental stressors, circadian rhythm disruption, nutritional challenges, and cosmic radiation exposure, all of which collectively contribute to elevated health risks.

### 4.1. Circadian Disruption and Fatigue

Among aircrew members, circadian rhythm disruptions trigger desynchronization of physiological processes and are associated with an increased prevalence of sleep disorders, adverse metabolic changes, and elevated cardiovascular risk [10,11]. Chronic fatigue and limited daylight exposure contribute to mood deterioration and depressive symptoms [12,13], while reduced sleep quality and irregular working hours result in diminished alertness and cognitive performance [14,15]. The biological basis involves disrupted circadian regulation of cortisol, melatonin, and core body temperature [25] with individual tolerance influenced by circadian clock gene variations (CLOCK, BMAL1, PER3) [26]. Chronic occupational stress further intensifies these effects by disturbing the hypothalamic–pituitary–adrenal axis, leading to flattened diurnal cortisol profiles and compromised recovery [27,28]. Recent evidence also confirms persistent trends of sleep disturbances and fatigue-related disorders among military aviators, underlining the chronic nature of circadian strain in this occupational group [29].

### 4.2. Nutritional Challenges in Aviation

Aviation personnel face significant nutritional challenges due to frequent time-zone changes, confined cabin environments, and restricted access to high-quality food [16,17].

These occupational factors result in irregular meal timing, excessive snacking, heightened caffeine consumption, insufficient fruit and vegetable intake, and elevated saturated fat consumption [16,17]. Female cabin crew demonstrate higher rates of gastrointestinal complaints and snacking behaviors [17], while preliminary research indicates elevated overweight prevalence and poor dietary adherence [18].

Meal timing misalignment with biological rhythms exacerbates jet lag symptoms and metabolic dysfunction [7,21]. This combination of irregular eating, circadian misalignment, and restricted food variety creates persistent nutritional strain in aviation workers.

Recent evidence confirms that circadian misalignment reduces leptin levels while elevating glucose and insulin, thereby promoting weight gain [26]. Shift workers exhibit significantly higher BMI and metabolic syndrome prevalence compared to day workers [30], with increased consumption of energy-dense, nutrient-poor foods during working hours [16]. Contemporary research has documented specific nutritional deficits in military aviation personnel [31] and identified acute metabolic changes in fighter pilots [32]. Studies of chronobiological eating patterns reveal that meal timing disruption significantly affects total caloric intake and macronutrient distribution [33,34], while unfavorable meal scheduling contributes to circadian syndrome development [30]. Collectively, these findings underscore the complex relationship between disrupted schedules, altered metabolism, and inadequate diet quality among aircrew.

Diet quality is further affected by cabin environmental conditions that alter taste and smell perception. Cabin conditions characterized by low pressure, low humidity, and persistent engine noise significantly impair taste and smell perception, particularly reducing sensitivity to sweet and salty flavors. Airlines compensate by increasing sugar and salt content in onboard meals to maintain acceptable flavor profiles, consequently elevating caloric content and reducing nutritional quality [35].

Research demonstrates that altitude conditions substantially compromise gustatory function [35], with dry cabin air reducing saliva production and impairing taste bud function [36]. Low humidity environments (10–15% typical in aircraft cabins) reduce olfactory sensitivity by 25% [37], given that smell contributes significantly to overall flavor perception.

Recent advances in sensory science research conducted under aviation and space-analog conditions have identified methodological approaches for testing food acceptance under altered environmental conditions [38]. Experimental studies employing virtual reality and microgravity body postures demonstrate measurable changes in food odor perception and affective responses [39].Cabin humidity modeling research provides practical parameters for environmental control systems [40], while extreme isolation studies confirm progressive olfactory and gustatory function impairment over extended periods [41].

These environmental and sensory factors not only alter the dining experience but may also affect food intake and nutritional quality, thereby contributing indirectly to metabolic and cardiovascular strain. Excessive sodium intake from compensatory food formulations increases hypertension risk [42,43], while nutritional recommendations emphasize sodium reduction rather than increased intake particularly among populations at elevated cardiometabolic risk. Meta-analyses have confirmed that a reduction of approximately 2.3 g of dietary sodium per day significantly lowers blood pressure in both hypertensive and normotensive individuals. Contemporary evidence demonstrates dose-dependent cardiovascular benefits of sodium reduction [42,43] and clinical efficacy of salt substitutes [44].

### 4.3. Cabin Environment and Respiratory Health

Aircrew encounter specific cabin environmental conditions that adversely affect respiratory health. Low humidity (approximately 16% ± 5%) promotes dehydration of mucous membranes, while reduced cabin pressure alters breathing mechanics and elevated ozone concentrations irritate the airways [45,46]. Recent measurements confirm that cabin air contains increased concentrations of ultrafine particles, which fluctuate with engine power and ventilation settings [46], and significant levels of volatile organic compounds including tetrachloroethylene from cleaning agents and cabin materials [47]. Contemporary research demonstrates that contaminated cabin air events expose aircrew to complex mixtures of organophosphates, volatile compounds, and nanoparticles, with systematic reviews documenting associated health consequences and recommending standardized medical assessment protocols [48]. Carbon monoxide levels in cabins have been analyzed with recommendations for enhanced monitoring and lower occupational exposure limits for aircrew [49].

These findings suggest that even short-term exposures may lead to mucosal irritation, reduced breathing comfort, and increased vulnerability to respiratory infections [50]. Aviation-related microbiome alterations may further compromise respiratory health through disrupted microbial balance [51].

### 4.4. Cancer Risk and Cosmic Radiation Exposure

Pilots and flight attendants experience chronic exposure to cosmic ionizing radiation (CIR) and ultraviolet radiation, which has been associated with an increased risk of certain cancers, particularly melanoma and breast cancer [52]. This occupational hazard is further intensified by long flight hours, high-altitude exposure, and disrupted circadian cycles.

In addition to radiation and circadian misalignment, light exposure during night operations may further elevate cancer risk through suppression of nocturnal melatonin secretion. Melatonin acts as an endogenous oncostatic, antioxidative, and immune-modulating hormone, and its reduction has been shown to facilitate tumor growth mechanisms, as extensively described by Blask [53].

Large cohort studies with cumulative dose assessment demonstrate signals for melanoma and brain tumors in pilots [54]. Recent measurements confirmsignificant UVA transmission through cockpit windows, contributing to pilot melanoma risk [55]. Experimental evidence indicates that simulated cosmic radiation exposure activates breast tumorigenesis pathways involving estrogen receptor alpha (ERα), estrogen-related receptor alpha (ERRα), and secreted phosphoprotein 1 (SPP1) signaling, thereby providing biological plausibility for observed epidemiological associations [56].

Prospective biomarker studies reveal suppressed DNA repair capacity in flight attendants after air travel, with diminished repair of radiation-induced damage and 8-oxoguanine:cytosine (8-oxoG:C)lesions [57]. Contemporary research highlights the peak cancer incidence typically occurs between ages 55–79 in men and 50–74 in women, making long-term follow-up essential for aviation workers who begin careers at young ages. Current evidence supports cosmic ionizing radiation, circadian rhythm disruption, and longer aviation career duration as probable contributors to elevated cancer risk [58].

An additional methodological consideration relevant to the interpretation of these health outcomes is the structural confounding between age, job tenure, and long-haul route exposure. Airline long-haul and trans-meridian operations are disproportionately assigned to more senior crew members, creating systematic overlap between older age, longer flight experience, and greater circadian stressors [59]. Although formal eligibility rules vary across airlines, operational data indicate that seniority and long-haul duty frequently co-occur, making it difficult to isolate the independent effects of time-zone transitions from accumulated exposure [59,60]. Recent operational studies demonstrate that age can moderate the association between night-flight duration and sleep disturbances, suggesting that biological ageing and occupational tenure may jointly influence vulnerability to circadian disruption [60]. To strengthen causal inference, future work should incorporate seniority metrics, route history, and longitudinal duty-pattern tracking to avoid misattributing circadian-related morbidity solely to chronological age [7].

### 4.5. Endocrine and Reproductive Health in Aircrew

Female flight attendants face significantly elevated risks of reproductive and endocrine complications linked to cosmic ionizing radiation, circadian rhythm disruption, and occupational stressors. Comprehensive epidemiological evidence demonstrates increased reproductive health risks among flight attendants. McNeely et al. reported elevated rates of infertility, miscarriages, preterm births, and fetal abnormalities compared to the general population [61] while Grajewski et al. specifically identified a correlation between cosmic radiation exposure levels and miscarriage rates [62].

Recent research reveals concerning impacts on ovarian function, with flight crew showing significantly lower anti-Müllerian hormone (AMH) levels and accelerated ovarian reserve decline compared to controls [63]. Hormonal disruptions include elevated prolactin levels predisposing to infertility and menstrual disorders, occurring independent of flight frequency in both short-haul and long-haul attendants [63]. Thyroid dysfunction manifests as elevated TSH levels (2.59 vs. 1.52 mcIU/mL in controls) and increased prevalence of thyroid antibodies, with hypothyroidism reaching 4.7% in long-haul attendants compared to general population rates [64,65]. These effects arise from multiple mechanisms including cosmic radiation-induced DNA damage, circadian disruption of hypothalamic-pituitary axes, melatonin dysregulation, and occupational stress-mediated HPA axis activation [62,63,66].

### 4.6. Gastrointestinal and Microbiome Considerations in Aviation Personnel

Aviation personnel experience significantly higher rates of gastrointestinal disorders and microbiome alterations linked to cabin environmental conditions, circadian disruption, and occupational stressors. Systematic reviews of shift workers and flight crews demonstrate increased prevalence of dyspepsia, irritable bowel syndrome (IBS)-like symptoms, constipation, and work-related fatigue compared to day workers, with strongest associations in night/rotating shift schedules and long-haul operations [67,68]. Reduced cabin pressure (equivalent to 2400 m altitude) causes intestinal gas expansion and gastric distension, leading to upper gastrointestinal discomfort and slowed gastric emptying during flight [69].

Hypobaric hypoxia exposure induces intestinal inflammation, oxidative stress, mucosal barrier disruption, and microbiome shifts toward reduced diversity and increased pro-inflammatory taxa including *Escherichia/Shigella*, *Blautia*, and *Dialister* [70,71]. Circadian misalignment from irregular schedules disrupts gut–brain axis function, altering microbial communities and promoting systemic inflammation that contributes to digestive complaints and metabolic dysfunction [72]. While direct evidence for increased antibiotic-resistant strain transmission in commercial aircrew remains limited, confined cabin environments and crew rest areas create conditions that may promote microbial exchange and opportunistic organism proliferation [73].

### 4.7. Integrated Cardiometabolic Risk and Organizational Determinants

Among the numerous occupational hazards confronting aircrew, cardiovascular diseases (CVDs) rank among the most prevalent, with risk strongly influenced by circadian rhythm disruption, occupational stress, prolonged sitting, and adverse cabin conditions [74,75,76].

Studies have shown that pilots are more often frequently overweight or obese (67% in New Zealand), physically inactive, exhibit insufficient fruit and vegetable intake, and sleep less than 7 h per day [75]. These lifestyle and occupational factors interact synergistically, thereby amplifying long-term cardiometabolic burden. Elevated CVD risk is also associated with impaired glucose and lipid metabolism, inflammation, and autonomic nervous system dysfunction. This risk increases after just 5 years of shift work, with an additional 7.1% increase for every subsequent 5 years [77].

The heightened susceptibility of pilots to CVDs is attributed to work characteristics involving high stress, prolonged immobility, and tasks requiring intense concentration [76]. Recent evidence shows that asymptomatic military aircrew demonstrate 12% prevalence of clinically significant coronary artery disease [78], while systematic reviews reveal high rates of cardiometabolic risk factors among airline pilots [75]. Contemporary meta-analyses demonstrate dose-dependent relationships between night shift work duration and cardiovascular events [79], with rotating shift patterns showing particularly adverse metabolic effects [80]. Organizational risk factors including irregular schedules and prolonged duty hours significantly affect aircrew health [81], while physical occupational exposures (cosmic radiation, pressure changes, noise) contribute additional health risks [82].

## 5. Strategies to Mitigate Risks: A Narrative Review

Addressing the specific health risks faced by airline personnel requires structured, evidence-based interventions. One such approach was demonstrated by Wilson et al. [83] in a 16-week personalized program for 125 pilots. The intervention, which included targeted changes in nutrition (e.g., increased fruit and vegetable intake, controlled portion sizes), sleep hygiene (e.g., reduced screen time, establishment of consistent sleep routines), and physical activity (individualized MVPA moderate-to-vigorous physical activity goals based on WHO guidelines: ≥150 min/week of moderate or ≥75 min/week of vigorous activity), resulted in significant benefits: an increase in cardiorespiratory fitness by 4.5 mL/kg/min, a decrease in body fat by 3.7%, and a reduction in systolic blood pressure by 8.1 mmHg. These outcomes highlight the feasibility and clinical relevance of tailored health-promotion programs in aviation settings. A structured overview of major occupational health risks and corresponding countermeasures is presented in Table 1, summarizing actionable strategies across circadian, nutritional, environmental, reproductive, and organizational domains.

### 5.1. Sleep and Circadian Rhythm Management

Evidence from Pereira et al. [84] on Norwegian Air Ambulance and Austrian Christophorus pilots showed that short naps of approximately 10 min effectively alleviate sleepiness and enhance alertness, whereas longer naps (>30 min) are counterproductive due to sleep inertia—a transient state of reduced vigilance and cognitive performance following awakening [85,86].

Melatonin supplementation has also demonstrated effectiveness in improving sleep quality and daytime functioning among shift workers [87,88]. Beyond regulating sleep, melatonin exhibits antioxidant, anti-inflammatory, and cardioprotective properties [89,90,91,92,93], with experimental studies demonstrating significant improvements in redox status and recovery in physically demanding occupations [91,92].

Randomized and clinical evidence demonstrates that melatonin improves sleep quality, cognitive performance, and multiple sleep parameters in shift workers, despite heterogeneity in dose and study design [94,95,96]. Its mechanisms extend beyond circadian alignment, encompassing anti-inflammatory and mitochondrial-protective actions that may also mitigate cancer risk [89,97]. Recent meta-analyses from 2023 further indicate that melatonin supplementation improves metabolic parameters such as insulin resistance and glycemic control, emphasizing its therapeutic potential beyond sleep regulation [98]. Systematic reviews recognize melatonin as one of the most widely recommended pharmacological tools to support night-shift adaptation and mitigate circadian desynchrony [99,100].

In operational contexts, comprehensive fatigue-management systems integrate melatonin use (0.5–3 mg, 30–60 min before sleep) with chronotherapy, restricting consecutive night duties (<3 nights), controlled morning light exposure (≈ 10,000 lux), and scheduled short naps or walking breaks during night operations [29,67,72,87,101].

In cases of sleep-disordered breathing, melatonin may serve as adjunctive therapeutic option in combination with diagnostic evaluation and targeted treatment [29,102]. Brief bouts of physical activity such as 5-min walking breaks every 30 min combined with light-based countermeasures effectively reduce operational sleepiness and maintain alertness [88,103].

### 5.2. Nutritional and Sensory Interventions in Aviation Personnel

Available evidence indicates that both the timing and composition of meals can significantly affect alertness, sleep quality, and metabolic homeostasis among shift workers. Dietary approaches such as adjusting macronutrient distribution (e.g., reducing carbohydrate intake at dinner), providing targeted snacks during extended night shifts, or restricting food intake to daytime hours have been shown to enhance alertness, reduce sleepiness, and stabilize mood [104,105,106].

Observational and field studies substantiate the real-world feasibility of meal-timing strategies [107,108]. De Rijk et al. demonstrated that the timing of macronutrient intake during night shifts significantly affects alertness levels, with carbohydrate consumption showing optimal effects when consumed 2–4 h into the shift rather than at shift onset [107]. Similarly, Flanagan et al. documented systematic redistribution of energy intake among rotational shift nurses, revealing that night-shift workers naturally adapt their eating patterns by concentrating caloric consumption during nighttime hours [108]. These adaptive dietary behaviors suggest that structured meal-timing interventions can be successfully implemented in real-world shift work environments. The evidence indicates that both the timing and macronutrient composition of meals during night shifts can be strategically optimized to enhance alertness and support circadian adaptation [107,108], while large cohort studies among flight personnel link late dinners and prolonged eating windows with increased risk of anxiety and depressive symptoms [21].

In addition to meal timing modifications, dietary enrichment with micronutrients essential for energy metabolism and oxygen transport, such as B vitamins, vitamin C, iron, magnesium, and zinc, also plays crucial role [109]. Supplementation with B-complex vitamins (particularly B1, B6, and B12) has been shown to enhance subjective energy levels [110], whereas iron deficiency even without anemia has been linked to fatigue and impaired performance [109].

In parallel, the unique sensory environment of the aircraft cabin influences food perception and requires specific dietary adaptations. Low pressure and humidity reduce taste and smell perception, necessitating food-product reformulation and multisensory design solutions. To offset the health risks associated with such sodium enrichment, potassium-based salt substitutes, gradual sodium-reduction programs, and routine blood-pressure monitoring are recommended [42,43,44]. Techniques such as umami enhancement, the addition of aromatic compounds, and virtual-reality-based acceptance testing have demonstrated effectiveness in maintaining palatability. Cabin humidity control at 40–60% further supports gustatory and olfactory function [39,40,111].

Pre-flight dietary counseling also addresses cabin-pressure-related gastrointestinal symptoms, recommending the avoidance of legumes and carbonated drinks, consumption of balanced meals 2–4 h before flight, and compliance with low-FODMAP principles [69,70].

### 5.3. Environmental and Engineering Controls

Environmental and engineering controls aim to minimize cabin air contamination, optimize air quality, and reduce occupational strain through technological and procedural innovation. To achieve these goals, standardized clinical and operational protocols have been established to investigate and manage aircrew exposure to contaminated cabin air events, including structured symptom assessment and biomonitoring where available [48]. Engineering interventions encompass upgraded ventilation and air conditioning (HVAC) filtration systems and continuous monitoring of carbon monoxide (CO) and volatile organic compounds (VOCs) in air supply channels [49]. Source control measures entail restricting high-emission cleaning agents and selecting low-VOC cabin materials based on source apportionment studies [47].

In parallel, ongoing technological upgrades such as real-time CO and VOC sensor systems in ventilation ducts and ergonomic improvements to galley design are being implemented to minimize environmental strain and improve crew comfort [112,113]. These initiatives are increasingly complemented by mindfulness-based stress-reduction programs for cabin crew [7].

Systematic review highlight humidity and temperature management, advanced filtration, and infection-control protocols as integral elements of occupational health protection [112].

### 5.4. Radiation Protection and Reproductive Health Surveillance

Comprehensive occupational health surveillance is essential for protecting reproductive and endocrine function in aircrew. Recommended monitoring procedures include baseline and periodic thyroid function testing (TSH, free T4, antibodies), ovarian reserve assessment (AMH, FSH) for female crew over 30 or with fertility concerns, and specialized reproductive counseling addressing occupational exposure risks [63,64,65].

As circadian misalignment contributes to endocrine and reproductive dysfunction, optimization of circadian health-through schedule control, limitation of consecutive night duties, light therapy, and supervised melatonin supplementation-can mitigate chronobiological disruption [66,93].

Complementary environmental and ergonomic interventions, including enhanced cabin air filtration systems, ergonomic workplace adjustments, and modified duty assignments during preconception and pregnancy, provide additional layers of physical protection [61,114]. Close coordination with reproductive endocrinologists and sleep medicine specialists ensures an integrated approach to managing the unique occupational exposure profile of aviation personnel [63,66].

Preventive strategies further encompass the installation of UV-shielding cockpit windows, routine use of sunscreen, and expanded cancer-screening protocols for high-exposure groups [52,54,55].

Finally, monitoring of DNA-repair biomarkers and implementation of targeted dietary interventions aimed at enhancing antioxidant defense mechanisms have been proposed as potential strategies to reduce molecular damage from chronic radiation exposure [56,58,83].

### 5.5. Gastrointestinal and Microbiome-Targeted Strategies

Comprehensive gastrointestinal health management in aviation combines organizational measures, dietary planning, and microbiome-targeted interventions. Optimizing duty schedules to minimize night-shift frequency, maintaining circadian stability, and implementing structured timed light exposure protocols can reduce the risk of digestive disturbances [67,72].

Dietary strategies should encompass time-restricted eating patterns, fiber-rich meals, attention to hydration and meal timing, while also prioritizing lower-gas-producing foods during flights to minimize pressure-related gastric distension [68,69].

Human randomized controlled trials indicate that Bifidobacterium supplementation may beneficially modulate gut microbial balance and improve psychological well-being under stress or irregular schedules, although direct evidence in occupational shift-work populations remains preliminary [115,116].

Evidence-based probiotic protocols often include *Bifidobacterium longum* JBLC-141 in combination with prebiotics such as inulin or fructooligosaccharides (FOSs) and fermented foods such as kefir or kimchi [67,70]. Enhanced cabin air quality management, regular health surveillance including gastrointestinal symptom screening, and referral to gastroenterology specialists for persistent symptoms provide additional clinical support [73]. Routine gastrointestinal monitoring using validated tools such as the Rome IV criteria, combined with quarterly microbiome profiling, may further enhance early detection and individualized management of digestive disorders in aircrew [68,72].

Research priorities include prospective aviation-specific cohort studies with validated symptom instruments, longitudinal microbiome sampling, and randomized trials of schedule interventions, targeted probiotics, and meal timing protocols to establish evidence-based prevention strategies [67,70,73].

### 5.6. Integrated Cardiometabolic and Organizational Health Strategies

Addressing the heightened cardiometabolic risk observed among aviation personnel requires an integrated approach that combines clinical surveillance, workload optimization, and targeted lifestyle interventions.

In high-risk aircrew, coronary computed tomography angiography (CCTA) is recommended form the age of 40 to facilitate early detection of subclinical coronary artery disease and to guide certification decisions [117]. Structured lifestyle programs integrating exercise, dietary counseling, and weight control have demonstrated substantial benefits in enhancing cardiorespiratory fitness and reducing blood pressure [83].

To counteract circadian disruption and metabolic dysfunction related to night and rotating shifts, optimized duty scheduling, fatigue-risk management systems, and chronobiological interventions such as strategic napping and circadian lighting are progressively implemented [79,80,81]. These strategies enhance sleep quality, metabolic stability, and operational performance.

Given the rising prevalence of obesity and diabetes among aircrew, comprehensive weight-management programs that integrate nutritional counseling, supervised physical activity, and, where indicated, pharmacological support can effectively improve body composition and glycemic control [76,111]. Occupational stress further intensifies cardiometabolic risk through neuroendocrine activation and behavioral pathways; therefore, workload modification, wellness initiatives, and ergonomic improvements are essential to reduce its impact [61,114].

Collectively, these multidimensional strategies illustrate the necessity of aligning individualized clinical care with organizational-level interventions to safeguard both cardiovascular health and operational safety in aviation professionals.

**Table 1:** Integrated mitigation strategies for aircrew: risk-intervention matrix.

Risk Factor	Mitigation Strategy or Intervention	Implementation	References
Sleep and circadian rhythm management			
Circadian disruption and shift work-related insomnia/fatigue	Melatonin supplementation, chronotherapy, strategic lighting, schedule adaptation to chronotype, fatigue risk management	0.5–3 mg 30–60 min before sleep; <3 consecutive night shifts; controlled light exposure (10,000 lux AM); strategic naps/walks	Carriedo-Diez et al. [87]; Caldwell and Knapik [29]; Boivin and Boudreau [118]; Lack et al. [101]; Kalra and Kour [119]; Grasa-Ciria et al. [67]; Khan et al. [72]
Oxidative stress and post-flight fatigue	Melatonin (antioxidant) + short walking breaks q 30 min during night shifts + performance recovery protocols	3–10 mg melatonin (antioxidant action)	Leonardo-Mendonça et al. [91]; Farjallah et al. [92]; Easton et al. [103]; Mahdi et al. [120]
Sleep apnea and breathing disorders	Melatonin (adjunct) + diagnostic and causal treatment + sleep hygiene education	3–6 mg melatonin (supportive)	Caldwell and Knapik [29]; Lasala and Lucero-Prisno [102]
Nutritional and sensory interventions in aviation personnel			
Irregular eating patterns/meal-timing misalignment	Chronobiological meal planning + structured schedules + education adapted to flight work	Timed meals aligned to circadian rhythms; pre-flight nutrition protocols	Carretero-Krug et al. [31]; Gonçalves et al. [33]; Silva et al. [34]
Nutritional deficiencies/poor food access	Targeted supplementation + improved in-flight catering + portable nutrient-dense packs	Vitamin D; micronutrient-controlled meals; access protocols	Gaździńska et al. [32]; Hemmer et al. [121]
Impaired taste and smell at altitude	Enhanced flavor profiling + multisensory food design + VR testing + controlled humidity	Umami enhancement; aromatic compounds; humidity 40–60%	Prabodha et al. [38]; Loke et al. [39]; Teleszewski and Gładyszewska-Fiedoruk [40]
Excessive sodium intake from flavor compensation	Salt substitutes + reduced-sodium formulations + blood pressure monitoring + CV risk assessment	Potassium-based salts; gradual sodium reduction; regular BP screening	Wang et al. [42]; Huang et al. [43]; Yin et al. [44]
Cabin pressure effects (GI gas and comfort)	Dietary modifications, gas-reducing foods, meal-timing optimization, pre-flight counseling	Avoid legumes/carbonated beverages; eat 2–4 h before flight; low-FODMAP education	Li et al. [70]; Qi et al. [69]
Environmental and engineering controls			
Cabin air contamination and chemical exposure	Contaminated-air event protocols + source control + continuous monitoring	Standardized medical investigation; low-VOC materials; CO/VOC sensors in air supply	Burdon et al. [48]; Dong et al. [47]; Hageman et al. [49]
Low humidity and particle exposure	Environmental controls + engineering solutions + personal protection	Humidity management; personalized ventilation; enhanced HVAC filtration	Georgescu et al. [113]; Michaelis et al. [46]; Wang et al. [42]
Environmental stressors	Advanced air quality systems + ergonomic galley design + stress management programs + crew rest optimization	HEPA upgrades; humidity 40–60%; mindfulness-based stress reduction	Qi et al. [69]; Bakr et al. [73]
Infection/resistance risk	Enhanced hygiene protocols + antimicrobial stewardship + surveillance systems + crew health monitoring	Hand hygiene > 95%; judicious antibiotic use; pathogen surveillance; regular health screenings	Bakr et al. [73]; Alyami et al. [68]
Radiation protection and reproductive health surveillance			
Cosmic radiation and UV exposure	Radiation dose monitoring + UV protection + enhanced medical surveillance + career exposure limits	Personal dosimetry; cockpit UV shields; sunscreen use; periodic cancer screening; rotation policies	Emslie et al. [55]; Scheibler et al. [52]; Dreger et al. [54]
DNA damage and impaired repair capacity	Biomarker monitoring + lifestyle interventions + preventive strategies	DNA repair capacity testing; antioxidant-rich diet; controlled interventions	Toprani et al. [58]; Kumar et al. [56]; Wilson et al. [83]
Cosmic radiation (exposure in pregnancy)	Dosimetric monitoring, route rotation, pregnancy duty modification	Personal dosimeters; polar route limits; modified assignments during pregnancy	Grajewski et al. [62]; Gómez et al. [122]
Thyroid dysfunction	Annual TSH + antibody testing + endocrine consultation	Baseline and periodic testing; specialist referral protocols	Radowicka et al. [65]; Chiovato et al. [64]
Ovarian reserve decline	AMH monitoring, reproductive counseling, fertility preservation	Testing for crew > 30 y; specialist consultation; early intervention	Barraza-Ortega et al. [63]
Gastrointestinal and microbiome-targeted strategies			
Microbiome dysbiosis	Probiotic therapy + prebiotics + fermented foods + microbiome monitoring	Bifidobacterium longum JBLC-141; inulin/FOS; kefir/kimchi; quarterly profiling	Li et al. [70]; Grasa-Ciria et al. [67]
GI symptom burden	Clinical assessment + symptom-directed therapy + occupational health coordination + validated tools	Rome IV criteria; IBS/dyspepsia management; GI referral; quarterly surveys	Alyami et al. [68]; Khan et al. [72]
Integrated cardiometabolic and organizational health strategies			
Cardiovascular disease risk (CAD, HTN, MetS)	Enhanced cardiac screening (CCTA) + risk stratification + lifestyle programs + medical certification guidelines	CCTA ≥ 40 y (high-risk); structured exercise; dietary interventions; updated certification criteria	Frijters et al. [78]; Kurek et al. [117]; Wilson et al. [83]
Shift work and circadian disruption (night shifts, irregular schedules, metabolic dysfunction)	Optimized work scheduling + fatigue risk management + chronotherapy + health monitoring	Limited consecutive night shifts; strategic napping; circadian lighting; regular assessments	Xi et al. [79]; Cho et al. [80]; Marqueze et al. [81]
Obesity and metabolic disorders (weight management, diabetes risk, inactivity)	Weight-management programs + pharmacological options + fitness protocols + nutritional counseling	Structured weight-loss programs; FDA-approved drugs (with safety evaluation); mandatory fitness standards	Prokop et al. [111]; Maculewicz et al. [76]
Occupational stress	Workload modification + stress-management + ergonomic improvements	Schedule adjustments; wellness programs; workplace modifications	McNeely et al. [61]; Kim et al. [114]

## 6. Discussion

Aviation personnel experience a complex interplay of occupational factors that collectively influence sleep, nutrition, metabolism, and overall physiological stability. Circadian misalignment defined as the mismatch between internal biological rhythms and external work schedules emerges as the primary pathophysiological mechanism linking irregular schedules with sleep disorders, metabolic imbalance, and nutritional dysregulation [7,10,11,16]. The aviation work environment characterized by time-zone transitions, confined cabin conditions, and irregular meal timing imposes cumulative strain on biological rhythms and dietary behavior. Consistent with prior research, our pilot findings confirm that sleep disturbances (71%) and fatigue (89%) are the most prevalent occupational health issues in this population, frequently accompanied by appetite and weight fluctuations, as well as limited access to nutritious food. These results are consistent with previous research showing that circadian misalignment and meal-timing irregularities profoundly alter metabolic and psychological outcomes among airline pilots and cabin crew [7,19,20,21].

Furthermore, circadian disruption is increasingly recognized as a key modulator of neuroendocrine signaling pathways, influencing cortisol secretion, insulin sensitivity, and energy metabolism in aircrew populations [27,79]. These data reinforce the need for targeted, aviation-specific fatigue-risk management strategies and nutritional interventions tailored to shift-working aircrew. Contemporary studies confirm that cardiometabolic risk factors among aircrew significantly exceed those of the general population, with pilots showing elevated rates of obesity, hypertension, and metabolic syndrome [78,117]. In our study, 60% of respondents reported weight changes after starting their aviation careers, suggesting long-term instability of energy balance and lifestyle adaptation. Increased appetite (47%) and decreased appetite (21%) were both common, reflecting dysregulation of hunger and satiety mechanisms caused by circadian misalignment [11,33,123]. Notably, 82% of participants were aged 18–35 years, suggesting early onset of metabolic and endocrine disturbances in a relatively young workforce. The dose-dependent relationship between shift-work duration and cardiovascular events underscores the cumulative nature of occupational health burden in aviation [79,81]. This highlights the importance of early preventive screening in young aviation personnel. Nutritional challenges extend beyond dietary restrictions, as cabin environmental conditions alter sensory perception, necessitating compensatory food formulations that contribute to excessive sodium intake and compromised nutritional quality [40]. Nearly two-thirds (63%) of participants reported altered taste perception typically describing reduced flavor intensity of inflight meals compared to those eaten on the ground which aligns with studies showing that low humidity and cabin pressure blunt gustatory and olfactory responses [36,37]. Environmental factors such as reduced cabin humidity (10–20%) and decreased air pressure have been shown to blunt sweetness and salt perception by up to 30%, while umami sensitivity remains relatively stable [35,36]. This phenomenon explains why inflight meals are often reformulated with higher salt and sugar content, a practice that, while improving palatability, may intensify cardiovascular and metabolic strain [42,43,124,125]. Chronobiological research highlights the importance of meal-timing alignment with circadian rhythms [33,67], and our data confirm frequent desynchrony between workday and off-duty meal schedules (>80% of respondents), reflecting circadian misalignment in dietary behavior.

Self-management practices emerged as compensatory strategies among aircrew. Melatonin use was reported by 41% of respondents, while 56% used dietary supplements most often vitamin D, omega-3 fatty acids, and magnesium. Although 76% of melatonin users reported improved sleep quality, these findings highlight a reliance on self-directed strategies in the absence of standardized institutional guidance. Similar patterns have been observed in previous research, demonstrating the growing popularity of chronobiological and nutraceutical approaches to counteract fatigue and circadian disruption [87,88,89,90,91,92,93,94,95,96,97,98,99]. High health literacy observed in our cohort reflects strong awareness of occupational risks, yet only half of participants reported adequate access to nutritious food during work. This discrepancy underscores a structural gap between knowledge and organizational support, emphasizing the need for airline-level nutritional and recovery standards [16,17,81]. Developing standardized educational programs on fatigue management, sleep hygiene, and evidence-based supplementation could improve both well-being and operational safety [83,87,88].

Female aircrew demonstrate concerning reproductive and endocrine health consequences, including increased thyroid dysfunction and higher risk of adverse reproductive outcomes [63,64,65,78]. Given that 63% of participants in this study were women, the results support prior evidence of occupational vulnerability among female flight attendants, especially those exposed to chronic circadian disruption and cosmic ionizing radiation [61,62]. Furthermore, thyroid dysfunction observed in this cohort may be partially mediated by circadian misalignment and altered melatonin–thyroid axis signaling [64,65]. These findings support the need for proactive reproductive counseling and periodic thyroid screening as part of standard occupational health programs alongside circadian-health optimization [61,62,81]. Environmental endocrine disruptors may further contribute mechanistically, though evidence is population-level rather than aircrew-specific [114,126].

The sex difference observed in our study, with 63% of women reporting symptoms, aligns with emerging evidence demonstrating that hormonal factors and circadian rhythm interact in sex-specific ways that may increase symptom prevalence in female aircrew [63,127,128]. Sex steroids, particularly estrogen and progesterone, exert modulatory effects on circadian clock function at multiple levels, including the suprachiasmatic nucleus, peripheral clocks, and clock gene expression [127,128]. These hormonal influences produce measurable differences in circadian physiology between women and men that extend beyond reproductive health.

Women exhibit earlier circadian timing and larger nocturnal declines in alertness compared to men, creating a physiological vulnerability to shift work and night-time schedules that characterize aviation operations [129]. Recent chronobiology data also show that circadian misalignment affects energy regulation differently in men and women, with women demonstrating greater metabolic sensitivity to misalignment [123]. Furthermore, women exhibit significantly greater light-induced melatonin suppression at bright illuminances (400–2000 lux), suggesting enhanced circadian photosensitivity that could amplify the disruptive effects of irregular light exposure during transmeridian flights [130].

Menstrual cycle phase introduces additional complexity, as fluctuations in estrogen and progesterone produce reproducible within-woman changes in circadian parameters. During the luteal phase, elevated progesterone increases nighttime core body temperature and alters circadian amplitude [131], while menstrual phase–dependent differences in neurobehavioral performance have been linked to the progesterone/estradiol ratio and core body temperature dynamics [131]. Women with premenstrual dysphoric disorder show altered circadian markers and sleep disturbances during symptomatic luteal phases [132,133]. These cycle-dependent variations may create temporal windows of heightened vulnerability to shift-work-related symptoms.

Female aircrew also exhibit higher circadian hormone rhythm amplitudes, with elevated plasma melatonin and cortisol area-under-curve values compared to men [134]. Hormonal contraceptive use—although primarily associated with thromboembolic risk—has been shown to modify cortisol dynamics and increase subjective fatigue in shift-working women, potentially influencing circadian-related symptom expression [125,135].

The convergence of sex-specific circadian architecture, menstrual-cycle effects, enhanced photosensitivity, metabolic sensitivity to circadian misalignment [123], and amplified hormonal rhythms provides a mechanistic framework for understanding why female aircrew may report symptoms at higher rates [63,123,127,128,129,130,131,132]. Epidemiological studies consistently show that women report more subjective sleep and circadian-related complaints than men, even when objective sleep measures are comparable or better [136]. In the context of aviation shift work, these sex-specific biological factors likely interact synergistically with occupational stressors—irregular schedules, transmeridian travel, and cabin environmental conditions—to amplify symptom burden in female personnel.

From an intervention perspective, comprehensive lifestyle programs have shown measurable benefits in aviation contexts, with significant improvements in cardiorespiratory fitness, body composition, and blood pressure among pilots [83]. Our findings, particularly the high prevalence of fatigue, nutritional challenges, and widespread melatonin use suggest that structured interventions combining circadian scheduling, nutritional education, and supervised supplementation could substantially improve crew well-being. The introduction of fatigue-risk management systems, controlled lighting exposure, and duty-rotation policies represents further actionable strategies [29,87,101,118]. Targeted modulation of the gut microbiome through fiber-rich, low-gas inflight meals may reduce gastrointestinal symptoms and stabilize metabolic responses under hypobaric and circadian stress conditions [67,70,115].

Circadian disruption has been linked to alterations in gut microbiome composition and inflammatory signaling, which may further contribute to metabolic instability among aircrew [67,70]. Given the emerging evidence on the gut–brain axis, dietary interventions that support microbial diversity may offer novel preventive avenue for shift-working populations [67,68,70].

This study has several limitations. The modest sample size (*n* = 101) and cross-sectional design restrict generalizability and causal inference. Data were self-reported, which may introduce recall bias, and participants represented multiple airlines and routes, contributing to heterogeneity in operational exposure profiles. The survey did not collect detailed shift-roster typology (e.g., fixed vs. rotating schedules; clockwise vs. counterclockwise patterns), which limits the ability to stratify participants by specific work schedules. Because the sample was recruited through purposive non-random sampling, selection bias cannot be excluded. In addition, the questionnaire was an ad hoc instrument developed for exploratory purposes, and although it underwent expert validation and pilot testing, it does not represent a standardized diagnostic tool. Items assessing new-onset symptoms over the course of an aviation career were interpreted descriptively and were not adjusted for age or duration of employment. Finally, the survey was intentionally designed as a single-cohort descriptive study without a control group, which limits comparative interpretation but is appropriate for a pilot investigation and will inform the design of future hypothesis-driven research.

However, the hybrid methodology—combining empirical data with a narrative review—allowed for the identification of specific, operationally feasible health-protection strategies tailored to aviation personnel. By integrating descriptive findings with multidisciplinary evidence, the study provides a coherent framework for understanding how circadian disruption, nutritional strain, environmental exposures, and endocrine factors jointly shape aircrew health. Table 2 summarizes key practical recommendations derived from these findings, outlining evidence-based strategies to improve health, performance, and safety in aviation personnel.

## 7. Conclusions

This study demonstrates that shift work exerts a substantial impact on the health and dietary habits of aviation personnel, with consequences extending beyond individual well-being and influencing overall operational safety. The observed prevalence of sleep disturbances, fatigue, and metabolic imbalance underscores the need for comprehensive occupational-health strategies tailored to this professional group.

Practical, evidence-based measures such as structured fatigue management through strategic napping, supervised melatonin use, balanced and circadian-aligned meals, regular physical activity, and preventive health screening can support physiological resilience and reduce occupational health risks. The development and implementation of such targeted interventions require coordinated efforts between aviation operators, occupational-health specialists, and regulatory bodies. Establishing standardized programs for fatigue management, nutritional support, and preventive monitoring will be essential to safeguard crew well-being and ensure long-term operational safety in the aviation environment.

## Figures and Tables

**Table 2:** Key findings and practical recommendations for aviation personnel.

Practical Recommendation	Expected Effect
Take short naps (10–20 min) during night or long flights; avoid naps > 30 min.	Improves alertness and reduces fatigue without post-nap grogginess.
Use melatonin (0.5–3 mg, 30–60 min before sleep) after night duties or long-haul flights.	Enhances sleep quality and circadian adaptation.
Follow balanced, daytime-aligned meals; choose fiber-rich, low-sodium foods, maintain hydration, and include fermented or probiotic-rich products.	Supports digestive comfort, microbiome balance, and metabolic stability.
Engage in ≥150 min of moderate or ≥75 min of vigorous activity per week.	Improves cardiovascular fitness and reduces metabolic risk.
Apply UV-protective sunscreen and eyewear; prioritize cabins with filtered, humidified air (40–60%).	Reduces radiation exposure and respiratory irritation.
Perform regular medical surveillance (thyroid, cardiovascular, and metabolic screening).	Enables early detection and management of occupational health risks.
Practice stress-reduction techniques and ensure recovery time post-duty.	Improves psychological resilience and overall well-being.

## Data Availability

The datasets generated and analyzed during the current study are available from the corresponding author on reasonable request. Data are not publicly available due to participant privacy protection and institutional data protection policies.
